# Epithelial-Mesenchymal Transition and Metabolic Switching in Cancer: Lessons From Somatic Cell Reprogramming

**DOI:** 10.3389/fcell.2020.00760

**Published:** 2020-08-06

**Authors:** Xiaowei Lai, Qian Li, Fang Wu, Jiechun Lin, Jiekai Chen, Hui Zheng, Lin Guo

**Affiliations:** ^1^CAS Key Laboratory of Regenerative Biology, Guangzhou Institutes of Biomedicine and Health, Chinese Academy of Sciences, Guangzhou, China; ^2^Bioland Laboratory (Guangzhou Regenerative Medicine and Health Guangdong Laboratory), Guangzhou, China; ^3^Savaid Medical School, University of Chinese Academy of Sciences, Beijing, China; ^4^Guangdong Provincial Key Laboratory of Stem Cell and Regenerative Medicine, Guangzhou, China

**Keywords:** EMT, cancer, reprogramming, energy metabolism, glycolysis, OXPHOS

## Abstract

Epithelial-mesenchymal transition (EMT) and its critical roles during cancer progression have long been recognized and extensively reviewed. Recent studies on the generation of induced pluripotent stem cells (iPSCs) have established the connections among EMT, energy metabolism, DNA methylation, and histone modification. Since energy metabolism, DNA methylation, and histone modification are important for cancer development and there are common characteristics between cancer cells and stem cells, it is reasonable to identify mechanisms that have been established during both reprogramming and cancer progression. In the current review, we start from a brief review on EMT and related processes during cancer progression, and then switch to the EMT during somatic cell reprogramming. We summarize the connection between EMT and metabolic switch during reprogramming, and further review the involvements of DNA methylation and cell proliferation. The connections between EMT and mesenchymal-epithelial transition (MET) and cellular aspects including DNA methylation, histone modification and energy metabolism may provide potential new targets for cancer diagnosis and treatment.

## Introduction

**FIGURE 1:**
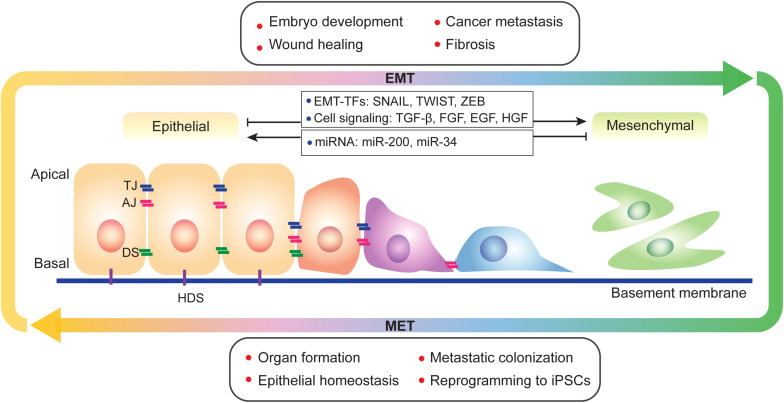
A brief view of EMT and MET. EMT and its reverse process MET occur during physiological and pathological progresses and display a spectral characteristic. During EMT, epithelial cells lost its apical-basal polarity and connections between cells and basement membrane. EMT is regulated by multiple factors, such as transcription factors, cell signaling and epigenetic modification. TJ, tight junction; AJ, adherens junction; DS, desmosome; and HDS, hemidesmosome.

Epithelial-mesenchymal transition (EMT) is defined as a biological process in which epithelial cells lose their characteristics and acquire mesenchymal features. During EMT, epithelial cells lose cell-cell junctions, apical-basal polarity, epithelial markers, and acquire cell motility, a spindle-cell shape, and mesenchymal markers. The concept of EMT was initially proposed as the epithelial-mesenchymal transformation by Elizabeth Hay in 1968 (Hay, 1968) as to describe the important cell changes in embryogenesis; it was later renamed EMT to distinguish it from neoplastic transformation (Nieto et al., 2016; Yang et al., 2020). EMT and its reverse process mesenchymal-epithelial transition (MET), display fundamental principles in diversified physiological and pathological progresses. During metazoan development, cells may sequentially undergo rounds of EMT and MET, as is seen in somite formation and heart development. EMT also occurs during wound healing in adults. Additional evidences related to development and wound healing has previously been reviewed extensively (Thiery et al., 2009; Lim and Thiery, 2012; Nieto et al., 2016). EMT also plays important roles in cancer progression and tissue fibrosis (Nieto et al., 2016; Pastushenko and Blanpain, 2019; Williams et al., 2019). Interestingly, during the processes of embryonic stem cells (ESCs) differentiation and induced pluripotent stem cells (iPSCs) formation, EMT, and MET are highly relevant to the loss and acquisition of pluripotency (Pei et al., 2019). EMT and MET are widely involved in various biological scenarios and display highly plastic and dynamic manners during cell fate transitions (Figure 1).

Epithelial-mesenchymal transition is regulated at different levels by multiple factors, including cell signaling, transcriptional control, epigenetic modification, and post-translational modifications (Figure 1). Epithelial cells receive EMT-inducing signals from their niches. For example, cytokines such as transforming growth factor-β (TGF-β), fibroblast growth factor (FGF) family, epidermal growth factor (EGF), and hepatocyte growth factor (HGF) can induce or promote the EMT process (Zavadil and Bottinger, 2005; Thiery and Sleeman, 2006; Nieto et al., 2016; Williams et al., 2019). These EMT-inducing signals up-regulate specific transcription factors called EMT-TFs (e.g., SNAI, TWIST, and ZEB; Thiery and Sleeman, 2006; Nieto et al., 2016; Williams et al., 2019). EMT-TFs usually cooperate with miRNAs as well as epigenetic and/or post-translational regulators to control EMT (Ye and Weinberg, 2015; Nieto et al., 2016). For example, the miR-200 family, which includes miR-200a, miR-200b, miR-200c, miR-141, and miR-429 play important roles in repressing EMT by repressing the translation of ZEB1 and ZEB2 (Korpal et al., 2008; Park et al., 2008). Moreover, overexpression of miR-200 family members can upregulate *E-cadherin* expression (Park et al., 2008). miR-200 family expression is regulated by DNA methylation; hypermethylation of miR-200 loci lead to the silencing of miR-200 family expression, which promotes the EMT process and causes human tumors (Davalos et al., 2012). The miR-34 family is also known as to regulate EMT and suppress early phases of tumor metastasis. Ectopic expression of miR-34a prevents TGF-β-induced EMT, and miR-34a/b/c regulate SNAI1 expression by its 3’-UTR. miR-34a also suppresses SLUG and ZEB1. Conversely, SNAI1, and ZEB1 also regulate miR-34a/b/c expression by binding their promoters (Siemens et al., 2011). Additional details on EMT regulation by miRNA have been reviewed in multiple papers (Zaravinos, 2015; Suzuki, 2018; Asadzadeh et al., 2019).

Metabolic changes occur during development and cancer progression. For example, both ESCs and tumor cells prefer glycolysis, this similarity between stem cells and cancer cells indicate the intrinsic mechanism of stemness maintenance and metabolism. More recently, metabolic pathways including glycolysis, the TCA cycle, and amino acid and lipid metabolism have been reported as being involved in EMT, especially in tumor progression (Kang et al., 2019; Georgakopoulos-Soares et al., 2020; Sun and Yang, 2020). During tumor progression, cells prefer to obtain energy by increasing glucose conversion into lactate, even in oxygen-rich condition (Fischer and Bavister, 1993; Zhou et al., 2012; Setty et al., 2016). This specific energy metabolism pathway is called aerobic glycolysis. It was first observed in tumors and described by Otto Warburg in the 1920s, and thus, was named the Warburg effect (Warburg, 1956). This metabolic reprogramming depends upon an increase in glucose uptake and highly activated glycolysis. Glucose transporters GLUT1 and GLUT3 can be induced by hypoxia-inducible factor 1α (HIF-1α), which contributes to glucose uptake and promote EMT and tumor progression (Macheda et al., 2005). Enzymes participate in glycolysis, such as hexokinase 2 (HK2), phosphofructokinase (PFK), and pyruvate kinase M2 (PKM2) participate in glycolysis, play positive roles in glycolysis flux, and induce EMT (Luo et al., 2011; Patra et al., 2013; Kim et al., 2017). Tumor cells also show abnormal lipid metabolism, such as increased lipogenesis (Swinnen et al., 2006). Enzymes that participate in lipogenesis, such as acetyl-CoA carboxylase (ACC), fatty acid synthase (FASN), and acyl-CoA synthetase long chain family member (ACSL), all show a close relationship with cancer and EMT (Georgakopoulos-Soares et al., 2020). For example, FASN, which synthesizes of palmitate from acetyl-CoA and malonyl-CoA, has been widely reported in various types of cancer. FASN reportedly promotes EMT through TGF-β signaling in lung cancer (Yang et al., 2016) and ErbB receptors in breast cancer (Zaravinos, 2015). Lipids are also important components of the plasma membrane. CTP-phosphocholine cytidylyltransferase (CTT), which is involved in phosphatidylcholine synthesis, is contributes to EMT in intestinal epithelial cells (Arsenault et al., 2013). Furthermore, amino acid metabolism is critical in EMT progression. Glutamine also plays important roles in energy supply. Glutaminase 1 (GLS1) and GLS2, which are involved in glutaminolysis can act as positive regulators of Snai1 (Choi and Park, 2018; Eiriksson et al., 2018). The important role of metabolism-related enzymes in cancer and EMT has resulted in many being selected as therapeutic targets. Additionally, metabolites from the pathways mentioned above can also regulate EMT through EMT-TFs and other epigenetic regulators, as we will discuss in this review.

This review discusses EMT progress and its regulators during cancer progression and iPSCs formation. We will also explore the relationship between metabolism and epigenetics in connection with EMT.

## EMT in Cancer and Stem Cells

### EMT Plays Critical Roles in the Cancer Metastatic Process

The important roles of EMT during tumorigenesis and metastasis have been demonstrated for decades. Most lethal human malignancies are derived from epithelial tissues, including the breast, colon, pancreas, and liver (Ye and Weinberg, 2015). Approximately about 90% of cancer-associated deaths are caused by metastatic disease rather than primary tumors (Lambert et al., 2017). The EMT program confers upon these epithelial cells properties critical to invasion and metastatic dissemination including notably increased motility, invasiveness, and the ability to degrade components of the extracellular matrix (ECM) components (Nieto et al., 2016). These complex metastatic cascades are orchestrated and coordinated by a series of master EMT-TFs that have been extensively explored (Craene and Berx, 2013; Lamouille et al., 2014). EMT-TFs facilitate the EMT process, but it is unclear whether EMT is indispensable for migration. Inhibiting EMT by overexpressing miR-200 did not affect lung metastasis when using an EMT lineage-tracing system in a spontaneous breast-to-lung metastasis model (Fischer et al., 2015). Furthermore, suppressing EMT by deleting *Snai* or *Twist* in a primary tumor did not decrease the invasion and metastasis of pancreatic carcinoma cells (Zheng et al., 2015). However, two subsequent have reports pointed out the shortcomings in these experiments (Aiello et al., 2017; Ye et al., 2017). These opposing opinions reflect the complexity of the EMT processes that must to be carefully investigated both *in vivo* and *in vitro*.

One of the difficulties in studying EMT arises because the transitions between epithelial and mesenchymal states are not binary. Instead, carcinoma cells often exhibit a spectrum of epithelial-mesenchymal characteristics (Jolly et al., 2017; Mathieu et al., 2017; Zhang and Weinberg, 2018). E-cadherin, occludins, and cytokeratins are the most commonly used markers for the epithelial traits, and N-cadherin and vimentin are the most commonly used for the mesenchymal state (Thiery et al., 2009). Recent studies have shown that some cancer cells, such as breast, pancreatic, renal, lung, and colorectal cancers, express both epithelial and mesenchymal markers (Bronsert et al., 2014; Sampson et al., 2014; Zhang et al., 2014; Schliekelman et al., 2015; Hiew et al., 2018). In fact, many cancer cells may not undergo a complete EMT process, but rather steadily acquire these intermediate, or hybrid epithelial/mesenchymal E/M phenotypes. The partial EMT or hybrid E/M phenotypes have multiple advantages compared to the complete EMT phenotype. It is suggested that these collectively migrating cells are the primary actors of metastasis (Jolly et al., 2015). To form distant metastases, cancer cells dissociate from the primary tumor, invade adjacent tissues, and intravasate into lymphatic and blood vessels to later colonize lymph nodes and distant organs (Nieto et al., 2016). Circulating tumor cells (CTCs) are defined as cancer cells that are released from a solid tumor and enter the peripheral blood; they are considered as biomarker of the metastatic process (Barcellos-Hoff et al., 2013). Most CTCs show hybrid E/M markers, suggesting an incomplete EMT. In addition, although EMT plays important roles in tumor progression, its reverse process, MET, also is significant in terms of dissemination. The last step of the invasion-metastasis cascade is termed colonization and largely depends on MET (Dongre and Weinberg, 2019).

### Relationship Between Cancer Stem Cell Plasticity, EMT, and Metabolic Reprogramming

The concept of cancer stem cells (CSCs) is based on the observation that not all cells in tumors are equal. A small number of tumor cells display two key features that distinguish them from the others: self-renewal and differentiation potential (Foster and Archer, 1979; Zhang and Wang, 2008). With these features, CSCs are hypothesized to play essential roles in tumor metastasis, heterogeneity, drug resistance, and recurrence. Since EMT and the metastatic cascade are inextricably linked, the relationship between EMT and CSCs has been investigated. A number of studies have now shown that EMT programs induce cancer cell stemness in many kinds of tissues (Dongre and Weinberg, 2019) and that EMT-TFs displayed the capacity to promote CSCs stemness in mouse and human models (Ye et al., 2015). Development of the cancer stem cell concept has been reviewed in several papers (Clevers, 2011; Batlle and Clevers, 2017). The isolation of CSCs from cancer cells and subsequent xenotransplantation assays provide evidence of the existence of CSCs. In the 1990s, CSCs were identified in acute myeloid leukemia (AML; Lapidot et al., 1994; Uckun et al., 1995; Bonnet and Dick, 1997). In 2003, CSCs were proved to be able to isolate from a solid tumor by a xenograft assay of small numbers of CD44^+^CD24^–/low^ cells which isolated from human breast cancer (Al-Hajj et al., 2003). Interestingly, cancer cells are observed exhibiting a plasticity that demonstrates the ability to switch between CSCs and non-CSCs in different cancers (Thankamony et al., 2020). For example, human basal breast cancer cells can switch from non-CSCs to CSCs by regulating the ZEB1 promoter (Chaffer et al., 2013). Another example for CSC plasticity is from studies on colorectal cancer. LGR5 is a marker of colorectal CSCs; a recent colorectal cancer study showed that LGR5 negative cells can become CSCs and lead to metastasis (Fumagalli et al., 2020).

Epithelial-mesenchymal transition-TFs are often found accompanied by features of stemness. *Slug* (also known as *Snai2*) is proved to be a key inducer of stemness (Guo et al., 2012; Nassour et al., 2012). Other EMT-TFs, such as *Twist*, *Snai1*, and *Six1* also induce stemness in various breast cancer models (McCoy et al., 2009; Morel et al., 2012; Ye et al., 2015). ZEB1 is required for stemness in pancreatic cancer; it inhibits members of the miR-200 family to maintain a stem-like phenotype (Wellner et al., 2009). EMT-related cell signaling factors, such as TGF-β, can be secreted in tumor stromal cells. This boosts metastatic potential and causes a poor-prognosis in colorectal cancer (Calon et al., 2015). Although the EMT process is associated with CSC characteristics, the relationship between EMT and stemness is still a controversial issue in tumorigenesis. Uncoupling EMT and stemness has been discussed in several studies and explained by the two events may occur in parallel (Nieto et al., 2016; Batlle and Clevers, 2017). EMT-TFs which critical in both EMT and CSCs are also provided evidence of uncoupling of the two events. For example, a study on Twist1 shows it is essential for initiate of skin tumorigenesis, however, Twist1 controlled tumor stemness independently of its EMT function (Beck et al., 2015). Recent studies have shown that cells with a hybrid E/M feature are much more likely to gain stemness (Bierie et al., 2017; Pastushenko et al., 2018). These studies may provide new insights for cancer therapy.

Metabolic reprogramming is one of the hallmarks of tumor progression. As discussed in the introduction, cancer cells change cell metabolism in diverse ways to gain energy and meet the requirements for proliferation. Glucose metabolism is considered the most studied metabolic change in CSCs. However, the glucose metabolism phenotype of CSCs, whether glycolysis or oxidative phosphorylation (OXPHOS), depends on both the tumor origin, and microenvironment (Thankamony et al., 2020). CSCs also show an interplay between glycolysis and OXPHOS (Yu et al., 2017). These metabolic characteristics increase the difficulty in providing effective cancer therapies; other metabolic pathways and metabolites must be investigated further.

### EMT-MET Processes in Physiological and Pathological Stem Cells

Stemness or stem cells are functionally defined as cells with the ability to self-renew and differentiate. In addition to cancer stem cells, other kinds of stem cells are isolated during development and in adults. One well accepted classification is that of ESCs and adult stem cells (ASCs). ESCs are derived from the inner cell mass of blastocysts and can differentiate into three germ layers and form chimeras (Evans and Kaufman, 1981; Martin, 1981), while ASCs are isolated from adult tissues in special niches and are capable of differentiating into several cell types depending on their origins (Shyh-Chang et al., 2013; Clevers, 2015). Although ESCs, ASCs, and CSCs are all considered as stem cells, their stemness is displayed in different ways. For example, both ESCs and ASCs show their differentiation abilities by forming normal tissues or cells. However, CSCs displayed their differentiation potential by promoting tumor heterogeneity (Clarke et al., 2006). These stem cells differ in terms of their gene expression profiles, transcriptional regulatory networks, and epigenetic modifications; however, they show their stemness in their consecutive EMT-MET changes. During embryonic development, early mesoderm cells are formed by EMT and migrate to generate intermediate mesoderm, chorda-mesoderm, paraxial mesoderm, and lateral plate mesoderm. These mesoderm cells then undergo MET to give rise to the urogenital system, notochord, somite, and somatopleure (Nieto, 2013). In contrast, CSCs caused by EMT undergo MET upon reaching an appropriate niche to become a new tumor (Nieto, 2013). Intriguingly, this EMT-MET progress has also been reported in pluripotency setup (Liu et al., 2013). Mouse embryonic fibroblasts (MEFs) can be reprogrammed into iPSCs by introducing exogenous *Oct4*, *Sox2*, *Klf4*, and *c-Myc*. Furthermore, reprogramming efficiency can be improved by sequentially expressing these transcriptional factors. All these phenomena imply that epithelial or even mesenchymal cells can reach a more active mesenchymal state to promote their plasticity.

## EMT in Reprogramming

### Early EMT Promotes Reprogramming

Direct reprogramming of somatic cells into iPSCs by defined factors is known as reprogramming (Takahashi and Yamanaka, 2006). A noticeable change that occurs during reprogramming is the transformation of MEF cells from a mesenchymal morphology into tightly packed colonies (Shu and Pei, 2014). This transition is defined as MET and is a critical step in acquiring pluripotency (Li et al., 2010; Samavarchi-Tehrani et al., 2010). Exogenous transcription factors *Oct4*, *Sox2*, and *c-Myc* are orchestrated to suppress *Snai1*, *Tgfb*, and *Tgfbr* to retain the non-mesenchymal characteristics of MEFs; *Klf4* induces epithelial properties by directly up-regulating *E-cadherin* (Li et al., 2010). Activating BMP signaling or repressing TGF-β signaling is beneficial for iPSC generation by promoting MET progress (Li et al., 2010; Samavarchi-Tehrani et al., 2010). Not long after the discovery of the essential role of MET during reprogramming, another publication revealed that a sequential EMT–MET mechanism at the beginning of reprogramming can enhancer reprogramming efficiency (Liu et al., 2013). Briefly, the sequential introduction of *Oct4* and *Klf4* first, followed by *c-Myc*, then *Sox2* (OK+M+S), can induce *Snai2* up-regulation and inhibit *E-cadherin* expression in the early stage of reprogramming. Further analysis revealed that this temporary EMT can generate iPSCs with a high efficiency of about 600% of basal level (Liu et al., 2013). Coincidentally, transient exogenous C/EBPα expression followed by the activation of Yamanaka factors (OSKM) in mouse primary B cells is capable of initiating EMT, which induces a 100-fold increase in reprogramming efficiency (Stefano et al., 2014). Hence, although MET is an essential event for reprogramming, having a more mesenchymal state at the early stage may provide a more facilitated state for cell fate transition.

### EMT and Metabolic Regulation Play Multiple Roles During Reprogramming

The generation of iPSCs is a multi-step process. In addition to MET, another important change is the metabolic switch that occurs during reprogramming. Unlike somatic cells that use OXPHOS to gain energy, pluripotent stem cells, including ESCs and iPSCs, and prefer glycolysis (Zhang et al., 2012). Using small molecules to activate glycolysis and inhibit OXPHOS or upregulate glycolytic gene expression can promote the metabolic switch from oxidative phosphorylation to glycolysis (OGS) and accelerate reprogramming (Folmes et al., 2011; Zhang et al., 2012; Prigione et al., 2014; Cao et al., 2015). Since both OKSM-induced EMT-MET processes and facilitated OGS accelerate reprogramming, it remains an open question whether EMT and OGS cooperate to regulate iPSCs.

**FIGURE 2:**
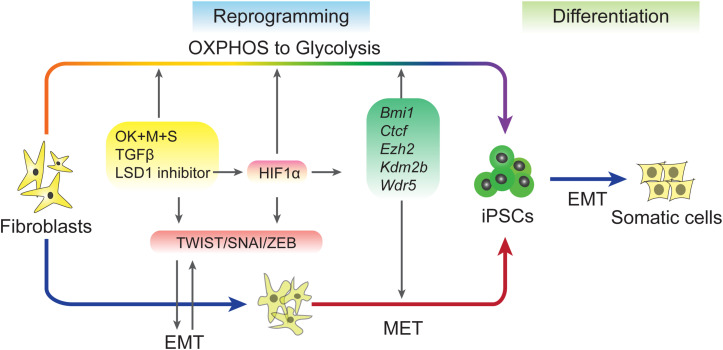
Regulation of EMT–MET during reprogramming. Induced pluripotent stem cells (iPSCs) can be accelerated by sequential EMT–MET process. Early EMT can be induced by sequential expression of Yamanaka factors, adding TGF-β or inhibiting of LSD1. These treatments up-regulation of EMT-TFs and also proved metabolic switch from OXPHOS to Glycolysis (OGS). EMT and OGS coordinate to facilitate the reprogramming through an epigenetic regulation way.

A recent study explored the relationship between early EMT and OGT (Sun et al., 2020). This study used 5C medium, a chemically-defined medium (He et al., 2015), to promote reprogramming by inducing a sequential EMT-MET process. Further analysis revealed that 5C medium increases glycolysis, inhibits OXPHOS, and accelerates the OGS process compared to a serum-containing medium. Early EMT and OGS may help cells overcome epigenetic barriers during reprogramming by upregulating five epigenetic factors, *Bmi1*, *Ctcf, Ezh2*, *Kdm2b*, and *Wdr5*, which have been proven to facilitate reprogramming in previous studies (Ang et al., 2011; Moon et al., 2011; Wang et al., 2011, 2017; Onder et al., 2012; Figure 2). The barriers that trapped cells at the pre-iPSC stage during reprogramming in serum containing medium (Chen et al., 2013b) may be overcome by the five epigenetic factors present in 5C medium. However, because the positive feedback loop between early EMT and facilitated OGS is too strong, further reprogramming is inhibited at the late stage by the induction of EMT and glycolysis to a level that is too high for further reprogramming and by preventing necessary MET (Sun et al., 2020). These findings are consistent other works showing that EMT induced at the late stage of reprogramming significantly impairs the generation of pluripotency (Liu et al., 2013). Furthermore, *HIF2α*, which is upregulated in 5C medium, represses reprogramming at later stages (Mathieu et al., 2014).

In addition to glycolysis, other metabolic pathways and metabolites are involved in EMT during reprogramming. A recent report showed that phospholipid remodeling is important for acquiring pluripotency by facilitating MET (Wu et al., 2019). This study demonstrated that the CDP-ethanolamine (CDP-Etn) pathway mechanically promotes reprogramming at the early stage through the CDP-Etn-Pebp1 axis to inhibit mesenchymal genes. Since metabolic regulation plays critical roles in EMT regulation and tumor progression, additional metabolic and EMT-MET connections in reprogramming need to be studied.

### Metabolism Regulates EMT and Reprogramming Through Histone Modification

As discussed above, while OGS upregulates five epigenetic factors in an HIF1α-dependent manner, early EMT promotes the expression of EMT-TFs that are enriched by both the five epigenetic factors and glycolytic genes (Sun et al., 2020). These reveal that the mesenchymal state may act as a link between metabolic and epigenetic regulation. Since metabolites generated during multiple metabolic processes are utilized in enzymatic reactions leading to epigenetic modifications and transcriptional regulation as previously reviewed (Ryall et al., 2015), we will focus on the crosstalk between EMT-MET, metabolism and epigenetic regulation during somatic cell reprogramming.

**FIGURE 3:**
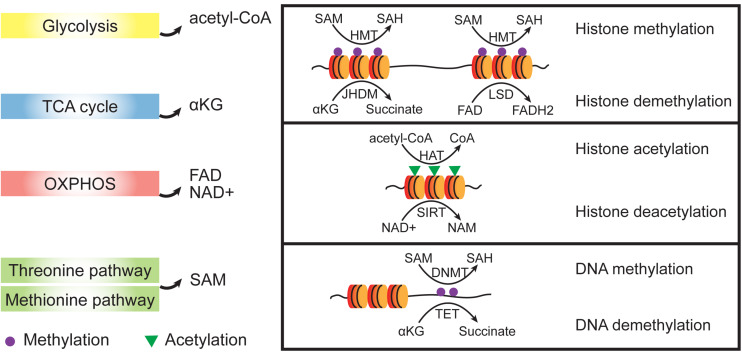
Crosstalk between metabolic and epigenetic regulation. Metabolites from different metabolic pathways participate epigenetic regulation as cofactors. Dioxygenases such as HMTs and DNMTs use SAM as methyl group donor and the reverse demethylation reactions use αKG or FAD to receive the methyl groups. Acetylation of histones is achieved by HATs which transfer acetyl groups from acetyl-CoA. To remove acetyl group from histone proteins, NAD+ is used as a cofactor to support the SIRT activities which belongs to histone deacetylases (HDACs). SAM, S-adenosyl methionine; SAH, S-adenosyl homocysteine; NAD+, oxidized nicotinamide adenine dinucleotide; NAM, nicotinamide; FAD, flavin adenine dinucleotide; αKG, α-ketoglutarate; HMT, histone methyltransferase; HAT, histone acetyltransferase; and SIRT, Sirtuin.

A vital role for metabolism in regulating cell fate has been derived from studies documenting rapid and dynamic changes in substrate utilization. Metabolites, including α-ketoglutarate (αKG), acetyl-coA, S-adenosyl methionine (SAM), and flavin adenine dinucleotide (FAD), regulate many of the important cell fate conversions by epigenetic catalytic reactions (Ryall et al., 2015; Wu et al., 2016; Figure 3). For example, glycolysis-produced acetyl-coA have been reportedly controls the early differentiation of ESCs by regulation histone acetylation (Moussaieff et al., 2015).

**FIGURE 4:**
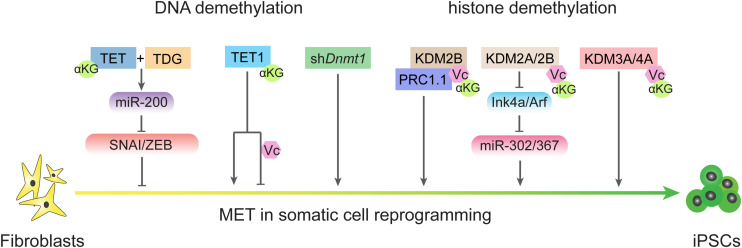
DNA demethylation and histone demethylation regulate MET process during reprogramming. TET and TDG are essential for early MET through demethylating miR-200 loci and TET1 plays different roles in reprogramming in condition of the present or absent of vitamin C. Passive DNA demethylation through knockdown the expression of Dnmt1 also promote MET. Histone demethylases also regulate MET process in different ways.

S-adenosyl methionine, which functions as a methyl donor for both DNA and histone methylation, is important for the maintaining of pluripotency in both mouse and human ESCs (Ng et al., 2013; Shiraki et al., 2014). The cellular αKG/succinate ratio contributes to the ability of ESCs to suppress differentiation (Carey et al., 2015). αKG/succinate ratios are involved in the regulation of a large family of αKG-dependent dioxygenases, such as Jumonji C (JmjC)-domain-containing histone demethylases (Carey et al., 2015; Figure 4). JHDM1A and JHDM1B (also known as KDM2A and KDM2B, respectively) belong to the Jumonji family of proteins and have been shown to demethylate H3K36me2/3 (He et al., 2008). Early studies showed that JHDM1A/1B inhibit Ink4a/Arf and activate miR-302/367 to promote reprogramming (Wang et al., 2011). A recent investigation showed that JHDM1B works as a component of the PRC1.1 complex and cooperates with BMP signaling to upregulate reprogramming efficiency by promoting MET (Zhou et al., 2017). Consistently, histone demethylases JMJD1 and JMJD2 (also known as KDM3A and KDM4A, respectively) function as the on/off switch for pre-iPSC fate by regulating H3K9 methylation status at core pluripotency loci (Chen et al., 2013b). In addition, lysine-specific demethylase 1 (LSD1, also known as KDM1A) functions through an FAD-dependent oxidative reaction to specifically catalyze the demethylation of H3K4me1/2 or H3K9me1/2 (Shi et al., 2004; Amente et al., 2013). Accordingly, inhibiting LSD1 by shRNA or a small molecular inhibitor can promote reprogramming at the early stage by increasing both OGS and exogenous transcriptional factors expression (Sun et al., 2016).

Histones can also be acetylated and deacetylated by histone acetyltransferases (HATs) and histone deacetylases (HDACs), respectively. HATs mediate the acetyl group transfer from acetyl-CoA, a metabolite from glycolysis. A higher level of acetyl-CoA is found in mouse ESCs compared to that of differentiated cells (Wang et al., 2020) and reportedly controls the early differentiation of ESCs by regulating histone acetylation (Moussaieff et al., 2015). In addition to histone acetylation, non-histone acetylation is also related to pluripotency. A recent study showed that the inhibition of CBP/EP300, which is closely related HATs, can repress the expression of mesenchymal transcription factor PRRX1 and promote reprogramming (Ebrahimi et al., 2019). HDACs are grouped into four classes. Class I (HDACs 1–3 and 8), class II (HDACs 4–7, 9, and 10), and class IV (HDAC 11) are zinc-dependent metalloproteins; class III (SIRT1-7) are NAD+-dependent proteins (Emmett and Lazar, 2019). Class I HDAC1-HDAC2 can form multiprotein complexes named SIN3A, NuRD, and CoREST, respectively. These HDAC containing complexes play different roles in establishing and maintaining pluripotency in a context-dependent manner. For example, the SIN3A/HDAC2 complex cooperates with NANOG to promote reprogramming (Saunders et al., 2017). However, HDAC2 reportedly acts as a barrier to reprogramming in other studies (Anokye-Danso et al., 2011; Wei et al., 2015). Interestingly, we noticed that miR-302/367 can promote reprogramming by inhibiting HDAC2 (Anokye-Danso et al., 2011), but in a contemporaneous study by Liao et al., miR-302/367 accelerated MET by inhibiting TGFBR2 and promoting E-cadherin expression (Liao et al., 2011). These studies indicate that the relationship between HDACs and EMT, and indeed, deacetylation by HDACs, can occur without histones. Snai1 interaction with HDAC1 and HDAC2 is dependent upon its SNAG domain and can mediate E-cadherin repression (Peinado et al., 2004). Sirtuins (SIRT1–7) belong to class III HDACs and depend on NAD^+^ to exhibit their enzyme activity. SIRT1 enhances reprogramming via SOX2 hypoacetylation (Mu et al., 2015), as well as through the miR34a-SIRT1-p53 axis (Lee et al., 2012). STRT7 reportedly reverse metastatic phenotypes in tumors (Malik et al., 2015) and may affect MET during reprogramming (Hsu et al., 2018).

### DNA Methylation Regulates EMT-MET During Reprogramming

DNA methylation is another area of crosstalk between metabolism and epigenetics that also plays critical roles in regulating EMT-MET during reprogramming (Figure 2). In mammals, methyl groups supplied by SAM are added at the 5-carbon of cytosines by the catalysis of DNA methyltransferases (DNMTs; Wu and Zhang, 2014). DNA methylation can be removed by both TETs-mediated active DNA demethylation and cell cycle-dependent passive DNA demethylation (Wu and Zhang, 2014, 2017). Since reprogramming occurs along with DNA demethylation in pluripotency-related loci, the mechanisms of DNA demethylation during iPSCs formation has been invested. TET proteins belong to αKG-dependent dioxygenases and require Fe^2+^ and vitamin C (Vc) as assistant (Ngo et al., 2019). Both TET1 and TET2 promote reprogramming (Doege et al., 2012; Costa et al., 2013; Gao et al., 2013), and under certain conditions, *Tet1* even could replace *Oct4* (Gao et al., 2013). Furthermore, active demethylation promoted by TETs and TDG is essential to reactive the miR-200 family that enables MET by inhibiting SNAI/ZEB in the reprogramming process (Hu et al., 2014). Repressing DNA methylation by sh*Dnmt1* or promoting passive demethylation by shp53-induced proliferation acceleration also promotes MET and facilitates iPSCs generation (He et al., 2017b, 2019). Further analysis has revealed that both sh*Dnmt1*-induced passive DNA demethylation and TET1-induced active DNA demethylation prefer hemi-methylated CpG sites that are enriched at the loci of core pluripotency genes and epithelial markers (He et al., 2019). The effects of DNA methylation on EMT-TFs were also studied in several types of cancer (Lee and Kong, 2016). Thus, metabolites from diverse metabolic pathways contribute to cell fate conversions by modulating epigenetic properties and show a high correlation with EMT-MET.

Intriguingly, further study of TET1 found that the physiological concentration of vitamin C increases TET1 activity, which in turn impairs the MET process and inhibits reprogramming (Chen et al., 2013a). In the absence of vitamin C, TET1 induces DNA demethylation on the loci of core pluripotency genes and epithelial markers rather than mesenchymal markers, which results in MET and accelerated reprogramming. The DNA demethylation activity of TET1 is enhanced in the presence of vitamin C. Because DNA methylation on the loci of core pluripotency genes and epithelial markers is already at a low level, the expression of core pluripotency genes and epithelial markers is not further affected. However, increased TET1 activity can induce DNA demethylation on the loci of mesenchymal markers, which in turn suppresses the expression of core pluripotency genes and epithelial markers. In addition to their DNA demethylation function, TET proteins also have important functions that are independent of catalytic activity. For example, TET1 could be recruited by Polycomb repressive complex 2 (PRC2) at H3K27me3 positive regions in ESCs (Neri et al., 2013). TET1 also interacts with SIN3A/HDAC complex (Vella et al., 2013) which is reported to promote pluripotency (Saunders et al., 2017). TET proteins including TET1, TET2, and TET3, are reported associated with the O-GlcNAc transferase (OGT) (Deplus et al., 2013; Vella et al., 2013; Chen et al., 2013c) in ESCs. OGT or O-GlcNAcylation could further recruit SET/COMPASS (Deplus et al., 2013) or modified transcription factors by O-GlcNAc, such as Pou5f1 (Constable et al., 2017), that may promote somatic cell reprogramming. Recent studies show that O-GlcNAcylation effects EMT through multiple pathways and promote cancer metastasis (Lucena et al., 2016; Harosh-Davidovich and Khalaila, 2018; Jiang et al., 2019; Feng et al., 2020), these may set up the relationship between TET and EMT. Tsai et al. reports TET1 could interacts with HIF-1α and HIF-2α to regulate hypoxia-induced EMT (Tsai et al., 2014).

Therefore, regulation of DNA methylation can affect both EMT and its reverse process, MET.

## Concluding Remarks

Although the field of EMT research has developed rapidly, many critical questions remain unanswered. For example, the driving force initiating EMT and the manner by which dose metabolism influences EMT, as well as the function of the mesenchymal state during the EMT-MET process are still unknown. Since transcription factors are important for cell identity, EMT-TFs are considered critical for losing epithelial characteristics and gaining mesenchymal phenotypes (Nieto, 2013). Hence, the regulation of EMT-TFs cooperating with cell signaling *in vitro* and transcriptional and epigenetic controls *in vivo* need to be carefully considered when studying EMT.

### Mesenchymal State Displays Plasticity of Cells

The concept of EMT comes from studies on early embryogenesis and is now widely investigated in cancer and stem cell biology. The transitions between mesenchymal and epithelial states demonstrate the plasticity of the cells. The sequential transition of EMT-MET not only occurs during tumor metastasis and somatic cell reprogramming as discussed above, but is also observed in cell differentiation and transdifferentiation. For example, a sequential EMT-MET drives the differentiation of human ESCs toward hepatocytes (Li et al., 2017). Activin A-induced formation of definitive endoderm (DE) accompanies a synchronous EMT mediated by autocrine TGF-β signaling and activates SNAI1 to initiate EMT followed by MET (Li et al., 2017). In addition, a temporary EMT-MET can increase the conversion efficiency of mouse astrocytes into induced dopamine neurons (Cervo et al., 2017) and human gastric epithelial cells into multipotent endodermal progenitors (Wang et al., 2016). Interestingly, in the early study of 5C medium, sequential EMT-MET was also observed during the transition of MEFs to neuron-like cells (He et al., 2017a). All of this evidence suggests that the mesenchymal state may be a necessary state for cell fate transition.

### Lessons From Somatic Cell Reprogramming Provide New Insights for Cancer Therapy

A recent study revealed the cooperation of EMT-MET and OGS during reprogramming as discussed above (Sun et al., 2020). However, coordinated regulation between EMT-MET and OGS should be investigated in other types of cell fate transition for common regulatory mechanisms. Metabolic shifts can control EMT during tumor metastasis, and metabolites from different metabolic pathways are involved in epigenetic regulation (Ryall et al., 2015; Kang et al., 2019). Both cancer cells and ESCs prefer to obtain energy through glycolysis, implying an intrinsic connection between the two kinds of cells. Furthermore, lessons can be learned from somatic cell reprogramming. For example, AML can be caused by the mutation of isocitrate dehydrogenase (IDH) IDH1 and IDH2 or TET2 (Figueroa et al., 2010; Quivoron et al., 2011). TET2 is a dioxygenase and its activity depends upon αKG, Fe^2+^, and Vc (Blaschke et al., 2013; Yin et al., 2013; Chen et al., 2013a). IDH1 and IDH2 convert isocitrate to αKG, which supports TET2 activity. A mutation in IDH1 or IDH2 causes the accumulation of 2-hydroxyglutarate (2-HG) and inhibits TET2 enzyme activity, resulting increased DNA methylation that drives AML progression (Lu et al., 2012). Previous studies on TETs in ESCs and reprogramming have showed that Vc can activate TETs as a cofactor, and a high dose of Vc has rescued TET2 deletion in a mouse model of leukemia (Agathocleous et al., 2017; Cimmino et al., 2017).

### Prospects for Cancer Therapy

Recent studies on CSCs focusing on their plasticity and resistance to therapy have been discussed in several other reviews (Batlle and Clevers, 2017; Najafi et al., 2019; Thankamony et al., 2020). Firstly, enzymes involved in metabolic reprogramming during tumor progression can be used as biomarkers for the therapeutic targeting of cancers (Sun and Yang, 2020). In addition to IDH, FASN in lipid synthesis and GLS1 in glutamine metabolism have been reported as targets for breast cancer and colorectal cancer, respectively, Sun and Yang (2020). Secondly, regulators involved in EMT can be used biomarkers and for therapeutic targeting (Williams et al., 2019). For example, both the transcriptional and epigenetic regulation of EMT-TFs could be treatment targets (Sun and Yang, 2020). Thirdly, since CSCs contribute to tumor resistance and recurrence, special attention must be paid to CSCs. Therefore, this review has discussed EMT in the context of both cancer and reprogramming, and explores the relationship between EMT, metabolism, and epigenetic regulation. These mechanisms could provide new scenarios for regenerative medicine and cancer therapy.

## Author Contributions

LG, XL, and QL wrote this manuscript. HZ, JC, FW, and JL helped to improve this manuscript. JC, HZ, and LG approved the final version of this submission. All authors contributed to the article and approved the submitted version.

## Conflict of Interest

The authors declare that the research was conducted in the absence of any commercial or financial relationships that could be construed as a potential conflict of interest.
